# Correlation of Morphological Appearance of Peritoneal Lesions at Laparotomy and Disease at Pathological Assessment in Patients Undergoing Cytoreductive Surgery for Peritoneal Malignancy: Results of Phase I of the PRECINCT Study in 707 Patients

**DOI:** 10.1245/s10434-024-16035-9

**Published:** 2024-08-26

**Authors:** Aditi Bhatt, Laurent Villeneuve, Armando Sardi, Amine Souadka, Alison Buseck, Brendan J. Moran, Basma El Khannousi, Carlos Gonzalez de Pedro, Dario Baratti, Danielle Biacchi, David Morris, Daniel Labow, Edward A. Levine, Faheez Mohamed, Gbadebo Adeleke, Gaurav Goswami, Isabelle Bonnefoy, Katherine Cummins Perry, Konstantinos I. Votanopoulos, Loma Parikh, Marcello Deraco, Mohammad Alyami, Noah Cohen, Nazim Benzerdjeb, Nehal Shah, Nezha El Bahaoui, Nazanin Khajoueinejad, Pascal Rousset, Perry Shen, Shoma Barat, Sophia Stanford, Selma Khouchoua, Samantha Troob, Sakina Shaikh, Umut Sarpel, Vadim Gushchin, Vasanth Mark Samuel, Vahan Kepenekian, Paolo Sammartino, Olivier Glehen

**Affiliations:** 1 Department of Surgical Oncology, Zydus Hospital, Ahmedabad, India; 2 Department of Clinical Research, Centre-Hospitalier Lyon-sud, Lyon, France; 3 Department of Surgical Oncology, Mercy Medical Centre, Baltimore, MD USA; 4 Department of Surgical Oncology, National Cancer Institute, Rabat, Morocco; 5 Department of Surgical Oncology, Peritoneal Malignancy Institute, Basingstoke, UK; 6 Department of Pathology, National Cancer Institute, Rabat, Morocco; 7 Department of Surgical Oncology, Hospital Universitario Virgen del Rocio, Sevilla, Spain; 8 Department of Surgical Oncology, Fondazione IRCCS Istituto Nazionale dei Tumori, Milan, Italy; 9 Department of Surgical Oncology, Sapienza University of Rome, Rome, Italy; 10 Department of Surgical Oncology, St. George Hospital, Sydney, NSW Australia; 11 Department of Surgical Oncology, Mount Sinai Hospital, New York, NY USA; 12 Section of Surgical Oncology, Wake Forest School of Medicine, Winston-Salem, NC USA; 13 Department of Pathology, Peritoneal Malignancy Institute, Basingstoke, UK; 14 Department of Radiology, Zydus Hospital, Ahmedabad, India; 15 Department of Pathology, Zydus Hospital, Ahmedabad, India; 16 Department of Surgical Oncology, King Khaled Hospital, Najran, Saudi Arabia; 17 Department of Pathology, Centre Hospitalier Lyon-sud, Lyon, France; 18 Department of Pathology, National Cancer Institute, Rabat, Morocco; 19 Department of Radiology, Centre Hospitalier Lyon-sud, Lyon, France; 20 Department of Clinical Research, Peritoneal Malignancy Institute, Basingstoke, UK; 21 Department of Radiology, National Cancer Institute, Rabat, Morocco; 22 Department of Surgical Oncology, Centre Hospitalier Lyon-sud, Lyon, Pierre Bénite France

**Keywords:** Cytoreductive surgery, Peritoneal cancer index, Surgical PCI, Morphology of peritoneal lesions, Peritoneal surface malignancy, Peritoneal metastases

## Abstract

**Background:**

The PRECINCT (Pattern of peritoneal dissemination and REsponse to systemic Chemotherapy IN Common and uncommon peritoneal Tumors) is a prospective, multicenter, observational study. This report from phase I of PRECINCT outlines variations in recording the surgical peritoneal cancer index (sPCI) at experienced peritoneal malignancy centers and the incidence of pathologically confirmed disease in morphologically different peritoneal lesions (PL).

**Methods:**

The sPCI was recorded in a prespecified format that included the morphological appearance of PL. Six prespecified morphological terms were provided. The surgical and pathological findings were compared.

**Results:**

From September 2020 to December 2021, 707 patients were enrolled at 10 centers. The morphological details are routinely recorded at two centers, structure bearing the largest nodule, and exact size of the largest tumor deposit in each region at four centers each. The most common morphological terms used were normal peritoneum in 3091 (45.3%), tumor nodules in 2607 (38.2%) and confluent disease in 786 (11.5%) regions. The incidence of pathologically confirmed disease was significantly higher in ‘tumor nodules’ with a lesion score of 2/3 compared with a lesion score of 1 (63.1% vs. 31.5%; *p* < 0.001). In patients receiving neoadjuvant chemotherapy, the incidence of pathologically confirmed disease did not differ significantly from those undergoing upfront surgery [751 (47.7%) and 532 (51.4%) respectively; *p* = 0.069]**.**

**Conclusions:**

The sPCI was recorded with heterogeneity at different centers. The incidence of pathologically confirmed disease was 49.2% in ‘tumor nodules’. Frozen section could be used more liberally for these lesions to aid clinical decisions. A large-scale study involving pictorial depiction of different morphological appearances and correlation with pathological findings is indicated.

**Supplementary Information:**

The online version contains supplementary material available at 10.1245/s10434-024-16035-9.

Three decades ago, Paul Sugarbaker devised the peritoneal cancer index (PCI) for quantifying the extent of peritoneal disease.^1^ The PCI has since proven to be a robust prognostic indicator for predicting the benefit of cytoreductive surgery (CRS) and is an important tool for therapeutic decision making.^2^ The PCI level is one of the factors considered while selecting patients for CRS for some primary tumors such as colorectal cancer, peritoneal mesothelioma (PeM), and gastric cancer, and by some surgeons for epithelial ovarian cancer (EOC); it is one of the variables used in predictive scores and nomograms, and a stratification factor in randomized trials.^3–9^

The PCI is essentially the surgical PCI (sPCI). Although it is recorded as a numerical value, there is more to it than just the absolute value. The sum of the lesion score (LS) in specific regions (e.g. the small bowel PCI) and the number of regions contributing to the sPCI, all have prognostic implications.^10,11^ Subsequently, the radiological PCI (rPCI) and pathological PCI (pPCI) were devised to document the disease extent on imaging and pathological evaluation, respectively.^12–14^

Although the sPCI is an objective variable, the evaluation of the disease extent by the surgeon is in itself a subjective evaluation. It has been shown that the sPCI differs from the pPCI in over 70% of patients.^14,15^ Surgeons tend to overestimate the disease extent more often than underestimating the extent. The accuracy also depends on the primary tumor site.^14^ In the era of precision medicine where multiple therapeutic modalities are available, a surgical intervention that provides no oncological benefit and carries a risk of morbidity should be avoided as far as possible, and a patient who could benefit from a procedure should not be denied the procedure. In this context, the importance of accurate prediction of the sPCI cannot be underestimated. A false positive assessment of the presence of disease could also lead to unnecessary resection of vital structures.

Arguably, of all oncological surgical procedures, CRS is the only procedure that is very heavily dependent on the surgeon’s visual inspection of the disease extent. Consequently, attention needs to be given to the morphological appearance of peritoneal lesions (PLs). To date, the morphological appearance of PLs remains a topic that has been poorly researched. There are a few studies that have looked at the incidence of microscopic disease in areas of post-chemotherapy scarring, and other studies that have described lesions as ‘suspicious’, ‘overtly malignant’, or ‘non-malignant’.^16,17^ We carried out a prospective observational study called PRECINCT (**P**attern of peritoneal dissemination and **RE**sponse to systemic **C**hemotherapy **IN C**ommon and uncommon peritoneal **T**umors) that involved a detailed recording of the sPCI, including the morphological appearance of PLs.^18^ This report from phase I of the PRECINT study looks at the variations in recording the sPCI at experienced peritoneal malignancy centers in different regions of the world and compares the pathological presence of disease in morphologically different PLs, as recorded by the surgeon.

## Methods

This was a prospective, multicenter, observational study. The detailed protocol of the study has been previously published.^18^ There was no therapeutic intervention in the study. The study comprised recording the demographic, clinical, radiological, surgical, and pathological details according to a prespecified format that included details of the distribution of disease, morphological appearance of peritoneal metastases (PM), regional node involvement, and pathological response to systemic chemotherapy (SC). All patients with biopsy-proven PM, from colorectal, appendiceal, gastric, or ovarian cancer, or PeM undergoing CRS with or without intraperitoneal chemotherapy were included. Written informed consent was obtained from all patients.

### Format of Data Collection

There are currently no reporting guidelines for both imaging and pathological evaluation of CRS specimens.^19^ The reporting format in this study included calculation of the rPCI and capturing other details such as the sites and structures bearing the largest tumor nodules and a description of the morphology in each region. Similarly, the surgical findings were to be documented in a systematic, prespecified manner. To ensure uniformity in the morphological description, a list of morphological features was made for the radiological, surgical, and pathological evaluations. This list was made in consultation with all the potential contributors and included radiologists and pathologists in addition to the surgeons. The PROMISE internet application was used as reference.^19^ The morphological description had to be provided with the LS for each region of the PCI. The first 6 months of the study comprised a test phase in which teams would assess the feasibility and compliance of this form of data capturing. This is a report from phase I. Phase II of the study is currently underway. During the second phase, there is an addition to the protocol for pathological evaluation in which pathologists will be required to take additional sections from the ‘normal appearing’ peritoneum adjacent to tumor nodules.

### Inclusion and exclusion criteria

- Only patients with biopsy-proven PM would be included (pathological evaluation was not mandatory prior to CRS if imaging or exploration confirmed the presence of disease).
- Patients undergoing second-look procedures with no evidence of PM were excluded.
- Patients undergoing debulking procedures were included.
- Patients undergoing palliative procedures that did not involve tumor debulking were excluded.
- For ovarian cancer, only patients with International Federation of Obstetrics and Gynecology (FIGO) stage III-C or IV-A were included. Patients undergoing surgery at first diagnosis, after neoadjuvant chemotherapy (NACT), and those undergoing surgery for recurrent disease were included. Patients without peritoneal disease were excluded.^18^

### Study endpoints

The primary endpoint is disease distribution in the peritoneal cavity, and the secondary endpoints are pathological response to SC, regional lymph node involvement, morphology of PM, overall survival (OS), and progression-free survival (PFS).^18^

### Sample Size

The number of patients required was determined based on our preliminary study looking at the disease distribution in relation to the disease extent. We considered involvement of different regions in relation to the sPCI as well as different structures in relation to the sPCI for each primary tumor. In addition, consideration was given to another endpoint, that is, pathological response to SC. This was only relevant for colorectal cancer and ovarian cancer. Details of the sample size calculation are provided in the published protocol: the target is 500 patients with colorectal cancer (350 post-NACT), 600 for ovarian cancer (350 undergoing interval CRS), 300 for appendiceal tumors and pseudomyxoma peritonei (PMP), and 100 each for gastric cancer and PeM.^18^

### Eithics

This study was approved by the Zydus Hospital (institution of the first author) Ethics Committee on 27 July 2020 (a specific study number was not provided by the committee). At Lyon-Sud Hospital, this study is being carried out within the framework of the RENAPE observational registry (CNIL-no. DR-2010-297) and the BIG-RENAPE registry (NCT02823860), Institutional Review Board (IRB) number A15-128.^20^ Subsequently, approval was obtained at other centers according to the existing institutional policies.

### Statistical Methods (for the Current Study Only)

Categorical data are described as number (%) and non-normally distributed continuous data are expressed as median (range). Categorical data were compared using the Chi-square test. For comparison of parametric and non-parametric data, the Student’s t-test and Mann–Whitney U test were used, respectively.

The list of terms for reporting the morphology of PM intraoperatively included ‘normal peritoneum’, ‘nodule’, ‘confluent nodules’, ‘plaque’, ‘thickening’, ‘scarring’, and ‘adhesion’. The use of other terms was permitted.

Lesions most likely to be malignant, such as ‘tumor nodules’, ‘confluent disease’, ‘mucinous deposits’, ‘omental cake’, and ‘plaques’, were termed *‘morphologically malignant lesions’*, while others that could be considered suspicious of harboring disease, such as ‘thickening’, ‘scarring’, and ‘adhesions’, were termed *‘morphologically suspicious lesions’.*

The incidence of disease on pathology for each morphological appearance was determined for each of the 13 peritoneal regions for every patient.

A similar comparison was made between ‘morphologically malignant’ and ‘morphologically suspicious’ lesions.

Based on the LS in regions with the morphological appearance of ‘tumor nodules’, these regions were divided into two groups—those with an LS score of 1 and those with an LS score of 2 or 3. The proportion of patients with disease confirmed on pathology in each group was compared.

## Results

From 15 September 2020 to 31 December 2021, 707 patients were recruited at 10 centers.

### Contributing Centers

Sixteen centers were considered for the study—2 index centers (of the first and last author) and 14 other centers. Three centers declined and three other centers volunteered to participate after publication of the protocol. Eventually, only 11/19 centers contributed to the study (two centers had problems with pathological evaluation and six centers could not complete other additional requirements for the study). Phase I had to be extended to 18 months to accommodate all the centers that wanted to contribute and to allow time for some centers to get ethical approval and other permissions in place. Data from one center is not included in this analysis due to missing data for all three PCIs.

The distribution of patients according to the center and primary tumor site is provided in electronic supplementary material (ESM) S1.

### Primary Tumor Site

The most common primary tumor site was ovarian cancer (*n *= 263), followed by appendiceal tumors and PMP (*n *= 181) and colorectal cancer (*n *= 153). NACT was used in 33 (86.8%) patients with gastric cancer, 107 (70.3%) patients with colorectal cancer, 171 (65.0%) patients with ovarian cancer, 19 (54.2%) patients with rare tumors, 8 (24.2%) patients with PeM, and 35 (19.1%) patients with appendiceal tumors and PMP. Secondary CRS was performed in 72 (10.1%) patients [57 (21.6%) ovarian cancer; 8 (4.3%) PMP; 3 (1.9%) colorectal cancer; 3 (8.5%) rare tumors, and 1 (3.0%) PeM]. Although it was not planned to include the uncommon primary sites or rare tumors, they were included since all the centers provided data for these tumors (ESM S2) .

### Surgical Peritoneal Cancer Index and its Additional Details

The sPCI is routinely reported at all 10 centers and was available for 683/707 (96.6%) patients. The median sPCI was 12 (range 0–39) [6 for gastric cancer (range 0–6); 7 for colorectal cancer (range 0–39); 12 for ovarian cancer (range 0–39); 16 for appendiceal tumors and PMP (range 0–39); 11 for rare tumors (range 0–30), and 13 for PeM (range 0–39)]. A completeness of cytoreduction (CC-0/1) resection was achieved in 683 (96.6%) patients.

In addition to the total value of sPCI, the description of morphological appearance, the structure bearing the largest tumor nodule, the size of the largest tumor nodule, and the LS for each region had to be captured. Of these four requirements, the LS was recorded at all centers and morphological details were routinely recorded at two centers, but all centers provided this detail for the study. The structure bearing the largest tumor nodule in each region was recorded at four centers and was available for the current study for only 50 patients. The exact size of the largest tumor deposit in each region was captured for selected patients at four centers.

### Peritoneal Regions Evaluated

**Fig. 1:**
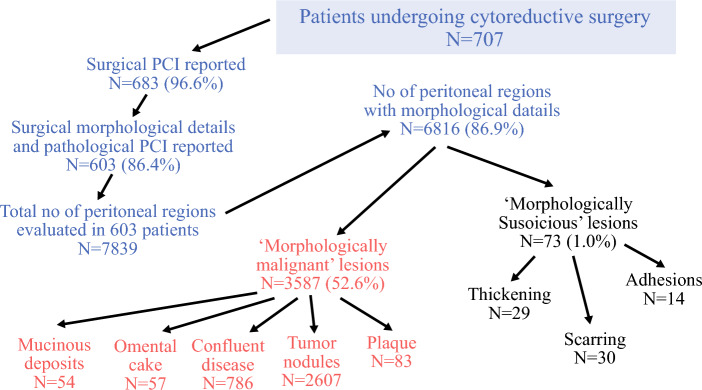
Synopsis of major findings of the study. *PCI* peritoneal cancer index

A synopsis of the main outcomes of this analysis is presented in Fig. 1. Both morphological appearance and pPCI were provided for 603 (86.4%) patients. Overall, 7839 regions were evaluable in 603 patients, of whom the morphological description was available for 6816 (86.9%) regions (Fig. 1). This discrepancy was due to missing details in some patients.

### Morphological Terms Used

In addition to the seven prespecified terms, six other terms were used (Table 1), amounting to a total of 13 terms. The most common morphological term used was normal peritoneum in 3091(45.3%) patients, followed by tumor nodules in 2607 (38.2%) patients, and then confluent disease in 786 (11.5%) patients. Prespecified terms were used in 6711 (98.5%) patients and unspecified terms were used in 105 (1.5%) patients.

**Table 1:** Prespecified and unspecified morphological terms used in 6816 regions evaluated in 603 patients

Morphological term	PCI region													Total [*N* = 6816] (%)
	0	1	2	3	4	5	6	7	8	9	10	11	12	
*Prespecified terms*														
Adhesion	0	2	1	1	0	0	1	3	2	0	0	1	3	14 (0.2)
Confluent disease	67	114	53	64	41	77	143	88	53	20	16	18	32	786 (11.5)
Normal peritoneum	166	178	262	254	272	179	121	192	213	325	332	328	269	3091 (45.3)
Omental cake	56	0	0	0	0	1	0	0	0	0	0	0	0	57 (0.8)
Plaque	12	15	6	8	11	9	6	6	5	2	0	0	3	83 (1.2)
Thickening	5	3	1	1	5	1	7	3	3	0	0	0	0	29 (0.4)
Scarring	2	5	1	2	3	2	4	3	4	1	0	0	3	30 (0.4)
Tumor nodules	236	201	182	175	193	240	241	217	234	160	166	164	198	2607 (38.2)
*Unspecified terms*														
Cyst	4	3	2	2	4	5	6	3	2	2	2	1	2	38 (0.5)
Scalloping	0	0	0	0	0	0	0	1	1	1	0	1	1	5 (0.07)
Mass	3	0	1	0	1	1	2	0	0	0	0	0	0	9 (0.1)
Mucinous deposits	4	4	6	4	5	5	3	3	4	4	3	3	6	54 (0.7)
Retraction	1	0	0	0	0	0	0	0	0	0	0	0	0	1 (0.01)
Granulations	0	1	0	0	1	0	0	1	2	0	0	0	0	5 (0.07)
Pseudonodules	1	1	0	0	1	0	0	1	1	0	0	0	0	5 (0.07)
Induration	0	0	0	0	0	0	0	1	0	0	0	1	0	2 (0.02)

*PCI* peritoneal cancer index

The use of different morphological terms according to the primary tumor site is presented in Table 2. The 13-region distribution of morphological terms for each primary tumor is provided in ESM S3–S8.

**Table 2:** Morphology of peritoneal lesions according to the primary tumor type

Morphological term	Colorectal cancer [*n* (%)]	Appendiceal tumors and PMP [*n* (%)]	Epithelial ovarian cancer [*n* (%)]	Gastric cancer [*n* (%)]	Peritoneal mesothelioma[*n* (%)]	Rare tumors [*n* (%)]	Total [*n* (%)]
Total no. of regions evaluated	1449 (21.2)	1663 (24.3)	2679 (39.3)	359 (5.2)	379 (5.5)	287 (4.2)	6816
Adhesion	2 (0.1)	2 (0.1)	10 (0.3)	0 (0.0)	0 (0.0)	0 (0.0)	14 (0.2)
Confluent disease	95 (6.5)	322 (19.3)	310 (11.5)	9 (2.5)	36 (9.4)	14 (4.8)	786 (11.5)
Normal peritoneum	870 (60.0)	560 (33.6)	1098 (40.9)	248 (69.0)	149 (39.3)	166 (57.8)	3091 (45.3)
Omental cake	11 (0.7)	7 (0.4)	35 (1.3)	0 (0.0)	3 (0.7)	1 (0.3)	57 (0.8)
Plaque	10 (0.6)	45 (2.7)	21 (0.7)	3 (0.8)	2 (0.5)	2 (1.0)	83 (1.2)
Thickening	14 (0.9)	6 (0.3)	8 (0.2)	0 (0.0)	1 (0.2)	0 (0.0)	29 (0.4)
Scarring	0 (0.0)	6 (0.3)	15 (0.5)	9 (2.5)	0 (0.0)	0 (0.0)	30 (0.4)
Tumor nodules	438 (30.2)	666 (40.3)	1168 (43.5)	85 (23.6)	161(42.4)	89 (31.0)	2607 (38.2)
Cyst	0 (0.0)	0 (0.0)	2 (0.07)	0 (0.0)	25 (6.5)	11 (3.8)	38 (0.5)
Scalloping	0 (0.0)	5 (0.3)	0 (0.0)	0 (0.0)	0 (0.0)	0 (0.0)	5 (0.07)
Mass	0 (0.0)	0 (0.0)	0 (0.0)	4 (1.1)	1 (0.2)	4 (1.3)	9 (0.1)
Mucinous deposits	9 (0.6)	44 (2.6)	0 (0.0)	1 (0.2)	0 (0.0)	0 (0.0)	54 (0.7)
Retraction	0 (0.0)	0 (0.0)	1 (0.03)	0 (0.0)	0 (0.0)	0 (0.0)	1 (0.01)
Granulations	0 (0.0)	0 (0.0)	5 (0.1)	0 (0.0)	0 (0.0)	0 (0.0)	5 (0.07)
Pseudonodules	0 (0.0)	0 (0.0)	4 (0.1)	0 (0.0)	1 (0.2)	0 (0.0)	5 (0.07)
Induration	0 (0.0)	0 (0.0)	2 (0.07)	0 (0.0)	0 (0.0)	0 (0.0)	2 (0.02)

*PMP* pseudomyxoma peritonei

### Classification of Morphological Appearances

**Fig. 2:**
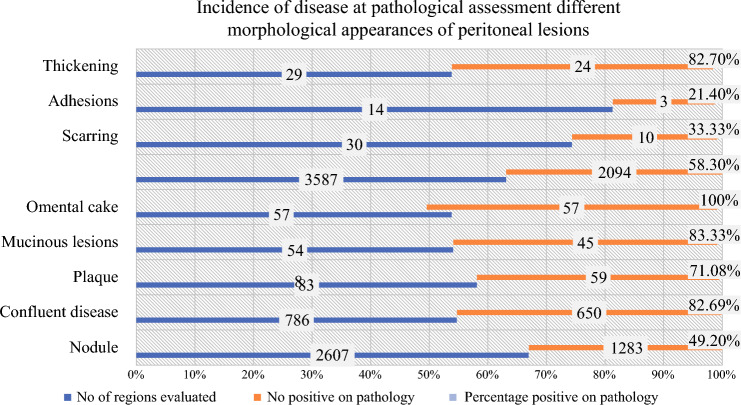
Incidence of disease at pathological assessment in different morphological appearances of peritoneal lesions

In total, 3587 (52.6%) lesions were classified as morphologically malignant and 73 (1%) lesions were classified as morphologically suspicious (Fig. 1). The use of both types of terms varied significantly according to the primary tumor type (Table 3). Both types of terms were used less for colorectal cancer, gastric cancer, and rare diseases, implying that the disease extent could be responsible for this difference. Among the morphologically malignant lesions, the incidence of disease on pathology was the highest for omental cake (100%), followed by mucinous lesions (83.3%), confluent disease (82.6%), and plaques (71.0%). Conversely, it was only 49.2% for tumor nodules. Of the morphologically suspicious lesions, presence of disease on pathology was highest in lesions labeled as ‘thickening’ (82.7%) and <40% for both scarring and adhesions. However, the incidence of disease in lesions termed ‘scarring’ was 33% and this term was most commonly used for ovarian cancer (Fig. 2). Overall, the incidence of disease on pathology was not significantly different among the morphologically malignant and morphologically suspicious lesions (58.3% vs. 50%; *p* = 0.260).

**Table 3:** Morphologically malignant and morphologically suspicious lesions according to the primary tumor site

Morphological term	Colorectal cancer [*n* (%)]	Appendiceal tumors and PMP [*n* (%)]	Epithelial ovarian cancer [*n* (%)]	Gastric cancer [*n* (%)]	Peritoneal mesothelioma[*n* (%)]	Rare tumors [*n* (%)]	Total [*n* (%)]	*p*-Value
Total no. of regions evaluated	1449 (21.2)	1663 (24.3)	2679 (39.3)	359 (5.2)	379 (5.5)	287 (4.2)	6816	
*Morphological appearance with high probability of disease (morphologically malignant)*								
Confluent disease	95 (6.5)	322 (19.3)	310 (11.5)	9 (2.5)	36 (9.4)	14 (4.8)	786 (11.5)	0.001
Tumor nodules	438 (30.2)	666 (40.3)	1168 (43.5)	85 (23.6)	161(42.4)	89 (31.0)	2607 (38.2)	0.001
Omental cake	11 (0.7)	7 (0.4)	35 (1.3)	0 (0.0)	3 (0.7)	1 (0.3)	57 (0.8)	0.040
Plaque	10 (0.6)	45 (2.7)	21 (0.7)	3 (0.8)	2 (0.5)	2 (1.0)	83 (1.2)	0.100
Mucinous deposits	9 (0.6)	44 (2.6)	0 (0.0)	1 (0.2)	0 (0.0)	0 (0.0)	54 (0.7)	< 0.001
Total	563 (38.8)	1084 (65.1)	1534 (57.2)	98 (27.2)	202 (53.2)	106 (36.9)	3587 (52.6)	0.001
*Morphological appearance that is suspicious of disease (morphologically suspicious)*								
Thickening	14 (0.9)	6 (0.3)	8 (0.2)	0 (0.0)	1 (0.2)	0 (0.0)	29 (0.4)	0.009
Scarring	0 (0.0)	6 (0.3)	15 (0.5)	9 (2.5)	0 (0.0)	0 (0.0)	30 (0.4)	< 0.001
Adhesion	2 (0.1)	2 (0.1)	10 (0.3)	0 (0.0)	0 (0.0)	0 (0.0)	14 (0.2)	0.166
Total	16 (1.1)	14 (0.8)	33 (1.2)	9 (2.5)	1 (0.2)	0 (0.0)	73 (1.0)	0.035

*PMP* pseudomyxoma peritonei

### Pathologically Confirmed Disease in ‘Tumor Nodules’

Overall, 1152 (44.1%) regions were given an LS of 1, and 1358 (52.0%) were given an LS of 2 or 3. The incidence of disease on pathology was significantly higher in regions with an LS score of 2/3 compared with an LS score of 1 (63.1% vs. 31.5%; *p* < 0.001). The 13-region distribution is provided in Table 4.

**Table 4:** Incidence of pathologically confirmed disease in lesions classified as tumor nodules stratified according totheir size (lesion score) and the use of neoadjuvant chemotherapy

PCI region	Morphological appearance of ‘tumor nodules’	Total number of pathologically positive lesions	‘Tumor nodules’ with LS 1^a^ [*n* (%)]	Pathologically positive LS 1 tumor nodules [*n* (%)]	‘Tumor nodules’ with LS 2/3^a^ [*n* (%)]	Pathologically positive LS 2/3 tumor nodules [*n* (%)]
All regions	2607	1222 (46.8)	1152 (44.1)	364 (31.5)	1358 (52.0)	858 (63.1)
0	236	117 (49.5)	58 (24.5)	21 (36.2)	162 (68.6)	96 (59.2)
1	201	111 (55.2)	57 (28.3)	16 (28.0)	138 (68.6)	95 (68.8)
2	182	89 (48.9)	89 (48.9)	25 (28.0)	89 (48.9)	64 (71.9)
3	175	77 (44.0)	70 (40.0)	22 (31.4)	100 (57.1)	55 (55.0)
4	193	78 (40.4)	79 (40.9)	20 (25.3)	107 (55.4)	58 (54.2)
5	240	141 (58.7)	86 (35.8)	39 (45.3)	142 (59.1)	102 (71.8)
6	241	138 (57.2)	70 (29.0)	23 (32.8)	169 (70.1)	115 (68.0)
7	217	106 (48.8)	90 (41.4)	40 (44.4)	108 (49.7)	66 (61.1)
8	234	112 (47.8)	98 (41.8)	23 (23.4)	120 (51.2)	89 (57.5)
9	160	56 (35.0)	108 (67.5)	35 (32.4)	50 (31.2)	21 (42.0)
10	166	58 (34.9)	117 (70.4)	32 (27.3)	43 (25.9)	26 (60.4)
11	164	56 (34.1)	108 (65.8)	25 (23.1)	52 (31.7)	31 (59.6)
12	198	83 (41.9)	122 (61.6)	43 (35.2)	78 (39.3)	40 (51.2)
*p*-value			< 0.001	< 0.001	< 0.001	< 0.001

PCI region	Morphological appearance of ‘tumor nodules’	Overall pathologically positive lesions [*n* (%)]	‘Tumor nodules’ in patients receiving NACT [*n* (%)]	Pathologically positive lesions after NACT [*n* (%)]	‘Tumor nodules’ in patients not receiving NACT [*n *(%)]	Pathologically positive lesions without NACT [*n* (%)]
0	236	127 (53.8)	142 (60.1)	75 (52.8)	94 (39.9)	52 (55.3)
1	201	111 (55.2)	116 (57.7)	60 (51.7)	85 (42.3)	51 (60.0)
2	182	100 (54.9)	110 (60.4)	58 (52.7)	72 (39.6)	42 (58.3)
3	175	84 (48.0)	115 (65.7)	59 (51.3)	60 (52.0)	25 (41.6)
4	193	78 (40.4)	114 (59.0)	40 (35.0)	79 (59.6)	38 (48.1)
5	240	141 (58.7)	142 (59.1	88 (61.9)	98 (41.3)	53 (54.0)
6	241	150 (62.2)	143 (59.3)	90 (62.9)	98 (37.8)	60 (61.2)
7	217	112 (51.6)	128 (58.9)	63 (49.2)	89 (48.4)	49 (55.0)
8	234	125 (53.4)	138 (58.9)	68 (49.2)	96 (46.6)	57 (59.3)
9	160	58 (36.2)	98 (61.2)	32 (32.6)	62 (63.8)	26 (41.9)
10	166	58 (34.9)	102 (61.4)	31 (32.3)	64 (65.1)	27 (42.1)
11	164	56 (34.1)	100 (60.9)	34 (34.0)	64 (65.9)	22 (34.3)
12	198	83 (41.9)	124 (62.6)	53 (42.7)	74 (58.1)	30 (40.5)
All regions	2607	1283 (49.2)	1572 (60.2)	751 (47.7)	1035 (39.8)	532 (51.4)

*LS* lesion score, *NACT* neoadjuvant chemotherapy, *PCI* peritoneal cancer index

^a^The LS was missing for some patients

We also studied the impact of NACT. Of the 2607 lesions classified as ‘tumor nodules’, 1572 (60.2%) were in patients who received NACT and 1035 (39.8%) were in patients who did not receive NACT. In patients receiving NACT, 751 (47.7%) lesions showed disease at pathological evaluation that did not differ significantly from those undergoing upfront surgery [532 (51.4%); *p* = 0.069] (Table 4).

### Normal Peritoneum and Incidence of Occult Disease

The term normal peritoneum was used for 3091 (45.3%) regions. The 13-region distribution is provided in ESM S9. Disease on pathology was observed in 335 (10.8%) regions. We did not distinguish between regions that were resected or not resected as this represents the normal course of clinical practice; this is elaborated in the Discussion section. The incidence of occult disease was highest in region 12 (24.5%) and lowest in region 11 (3.0%). The 13-region distribution of occult disease is presented in ESM S10.

## Discussion

This study illustrates the heterogeneity in recording the sPCI at experienced peritoneal malignancy centers around the world and highlights the need to further define and standardize the recording of this important prognostic indicator. Six centers could not contribute as they could not provide a detailed sPCI. The most significant finding was that only 50% of the lesions classified as ‘tumor nodules’ showed disease at pathological evaluation. The discrepancy was higher for smaller lesions (LS 1) (Table 4). This calls for more liberal use of frozen section to confirm the presence of disease in small tumor nodules. There was no difference in the incidence of pathologically confirmed disease in tumor nodules among patients who received NACT and those who did not. It might be expected that the accuracy of visual inspection would be higher when surgery is performed upfront, however this was not the case with ‘tumor nodules’. During upfront surgery, there could be confusion with benign lesions or inflammatory changes. Although unspecified terms were used in a small percentage of patients, these could be the lesions that require extra caution when deciding whether to resect the given area or not. In addition, 82.7% of the lesions classified as ‘thickening’ had disease at pathology.

**Fig. 3:**
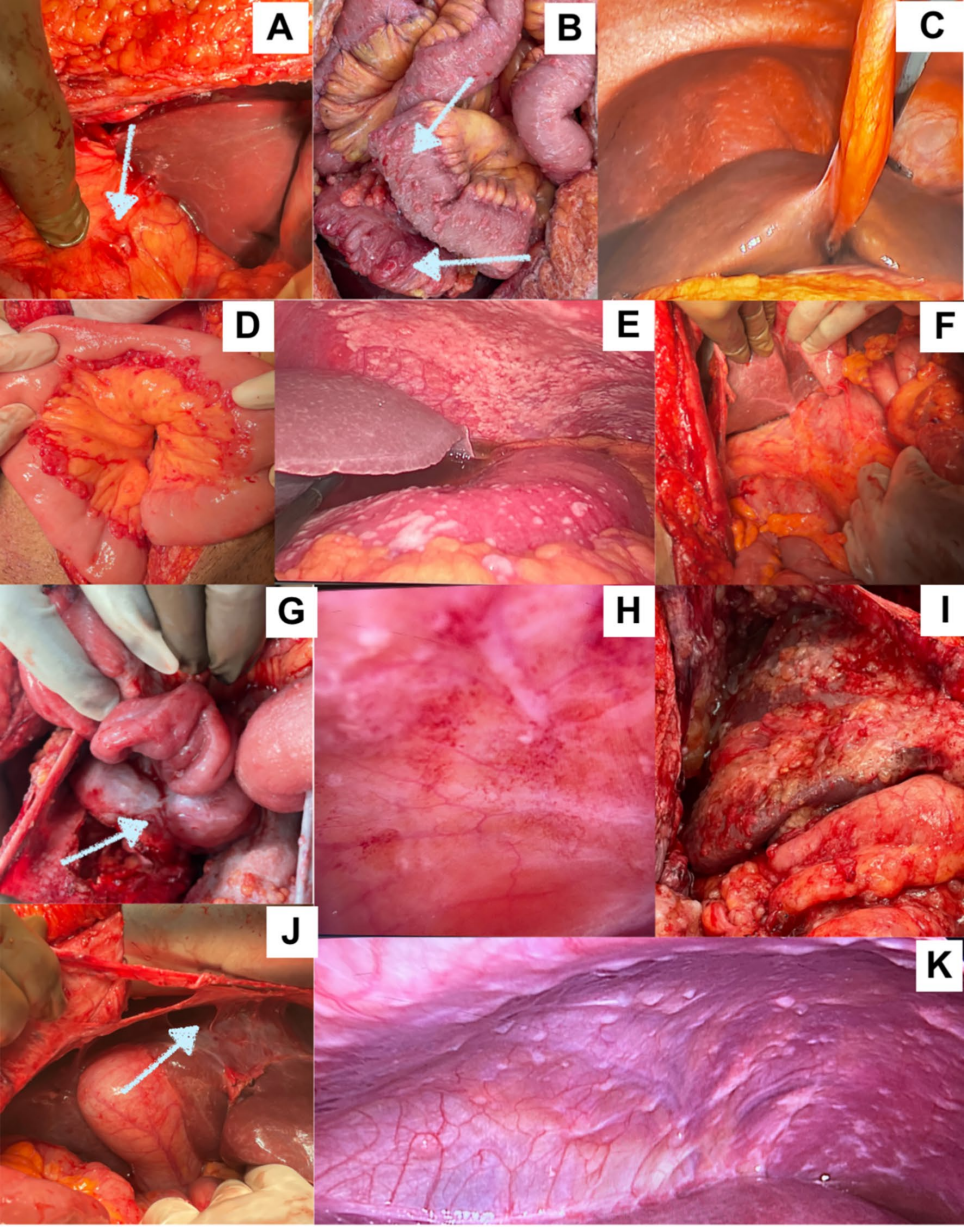
Different morphological appearances of peritoneal lesions. (**a**) Tumor nodule in ovarian cancer, pathologically positive. (**b**) Tumor nodules in PMP, pathologically negative. (**c**) Tumor nodules in ovarian cancer, pathologically positive. (**d**) Confluent disease in high-grade PMP, pathological positive. (**e**) Plaques and confluent disease in gastric cancer, pathologically positive. (**f**) Scarring in ovarian cancer, pathologically positive. (**g**) Adhesions in high-grade PMP, pathologically positive. (**h**) Thickening in ovarian cancer, pathologically positive. (**i**) Mucinous lesions in low-grade PMP, pathologically positive. (**j**) Adhesions in ovarian cancer, pathologically negative. (**k**) Nodules and thickening in sigmoid colon cancer, pathologically positive. *PMP* pseudomyxoma peritonei

The interobserver variation in recording the PCI has never been studied systematically.^21^ A large-scale study that involves pictorial depiction of different PLs and correlation with the presence of disease on pathology is needed to improve the accuracy of the sPCI in predicting the true extent of disease and in comparing outcomes from different centers. Examples of different morphological appearances is provided in Fig. 3.

### Tumor Nodules Could be Classified as ‘Morphologically Suspicious’

While only 50% of tumor nodules had disease at pathological assessment, in all other morphologically malignant lesions the incidence was more than 70%. To the best of our knowledge, this is the first study in which the morphology of PL was reported using a prespecified set of terms. In the study by Costantini et al., visual appearance of diaphragmatic disease was classified as visually pathologic or visually dubious.^17^ Over 80% of patients with visually dubious lesions had disease on pathology, while 100% of the visually pathologic lesions had disease on pathology. From the images presented in the manuscript, many surgeons could have classified the visually dubious lesions as pathologic.

While designing this study, we had no supportive studies for a baseline reference. Therefore, we could not predict what proportion of patients with ‘tumor nodules’ might have pathologically confirmed disease. A recently published manuscript evaluating the role of artificial intelligence in correctly distinguishing malignant from non-malignant lesions reported a similar incidence of disease at pathology (50%) when the evaluation was performed by surgeons.^22^

### Potential Role of Fluorescence-Guided Surgery

Another alternative method of classifying PL accurately is the use of fluorescence-guided surgery (FGS). Currently, large-scale validation studies on the role of FGS are lacking, as is standardization regarding the dye to be used, the timing of injection, and the imager to be used.^23,24^ The main advantage seems to be the high negative predictive value of 90–100%.^23^ Some of the dyes that are used, such as indocyanine green, detect lesions based on the vascularity, which may not be ideal for detecting malignant PLs, while others such as 5-aminolevulinic acid (ALA) are expensive and not widely available.^25,26^ Other targeted probes are being developed based on the molecular profiles of tumors.^27^ The performance of these in relation to tumor heterogeneity, timing of PM, the sites that are biopsied, and timing of surgery and the non-flourescent sites that are biopsied will need to be considered.^28^

### ‘Normal Appearing’ Peritoneum

While estimating the sPCI, even regions that are not involved or resected are evaluated, which is why the most common morphologic term used was ‘normal peritoneum’. The incidence of occult disease in lesions termed ‘normal peritoneum’ was 10%, which is in line with previously published reports.^19^ We did not make a distinction between regions that were or were not resected as this captures the clinical scenario at CRS and the incidence of occult disease could be higher. Some structures such as the greater omentum are removed in the majority of patients and the lesser omentum is removed in selected patients, even if not directly involved by disease, which explains why normal peritoneal regions were resected in this study.

### Variation of Morphological Terms According to the Primary Tumor Type

There was a significant difference in the morphological terms used for different diseases—normal peritoneum was reported in nearly 70% of the regions in patients with gastric cancer (median sPCI-6), but was reported for only 33% of patients with appendiceal tumors and PMP (median sPCI-16) (Table 2). Apart from the extent of disease itself, the reason for the variation could be that different primary tumors produce morphologically different lesions, for example, mucinous lesions in PMP, scarring in ovarian cancer after NACT, plaques in mesothelioma and signet ring cell tumors, and this will be studied in greater detail when the study completes recruitment. The mechanisms of peritoneal spread could have an impact on the morphologic appearance.^29,30^ Trans-lymphatic spread and hematogenous spread are likely to produce more diffuse involvement and miliary disease compared with transcoelomic spread, however this is an unproven assumption.

### Future Directive

The main takeaway from this study is that caution should be exercised when resecting regions that the surgeon classifies as ‘tumor nodules’. Frozen section could be used more liberally in these cases before performing potentially morbid procedures such as visceral resections or diaphragmatic stripping more so in older and frail patients. The same should be applied to ‘morphologically suspicious lesions’ such as ‘adhesions’ and ‘scarring’. Using pictorial depiction of various lesions to obtain a consensus among surgeons regarding the most appropriate morphological term for each one, it may be possible in future to limit the number of terms used. It could also help less experienced surgeons in intraoperative decision making.

### Strengths and Limitations

The biggest limitation of this study is the missing data. This was something that we had anticipated while designing the study. The study received no external funding and it was not possible to employ additional personnel for data collection, with the existing staff already working at full capacity. No additional training was organized for data collection as most of the information specific to this study was related to the three PCIs and required additional work from the surgeons, radiologists and pathologists. This was quite time-consuming for the radiologists and pathologists, which explains the 10–25% of missing data for many variables. The strengths of this study are its prospective nature and the involvement of high-volume centers from around the world, some of which have been pioneers in this field.

## Conclusions

The sPCI was recorded with a lot of heterogeneity in the details captured at experienced peritoneal malignancy centers. The incidence of pathologically confirmed disease was only 49.2% in lesions labeled as ‘tumor nodules’. A large-scale study that involves pictorial depiction of different morphological appearances of PLs and correlation with the presence of disease at pathological evaluation is needed to improve the accuracy of the sPCI in predicting the true extent of disease. Frozen section should be used more liberally in lesions classified as ‘tumor nodules’, and morphologically suspicious lesions such as ‘scarring’ and ‘adhesions’, to guide intraoperative decisions.

## Supplementary Information

Below is the link to the electronic supplementary material.

Supplementary file1 (DOCX 23980 KB)
