# Supplementary material for: Correlation of Morphological Appearance of Peritoneal Lesions at Laparotomy and Disease at Pathological Assessment in Patients Undergoing Cytoreductive Surgery for Peritoneal Malignancy: Results of Phase I of the PRECINCT Study in 707 Patients
Source: Ann Surg Oncol. 2024 Aug 26;31(13):8560–71. doi: 10.1245/s10434-024-16035-9 (PMC11549162; doi:10.1245/s10434-024-16035-9)

**Supplementary material**

Supplement S1 No of patients recruited in phase 1 at different centres stratified according to the primary tumour site

| **Centre /city country** | **Colorectal cancer** | **Appendiceal and PMP** | **Ovarian cancer** | **Gastric cancer** | **Peritoneal mesothelioma** | **Rare tumors** | **Total N (%)** |
| --- | --- | --- | --- | --- | --- | --- | --- |
| Lyon-sud hospital, Lyon, France | 90 | 50 | 119 | 25 | 13 | 22 | 319 (45.1) |
| Zydus, Ahmedabad, India | 3 | 14 | 90 | 1 | 5 | 4 | 117 (16.5) |
| NCI, Milan, Italy | 13 | 39 | 6 | 1 | 11 | 0 | 70 (9.9) |
| Sapienza hospital, Rome, Italy | 16 | 3 | 23 | 6 | 1 | 1 | 50 (7.0) |
| Mercy Medical centre, Baltimore, USA | 2 | 16 | 3 | 0 | 0 | 4 | 25 (3.5) |
| Wakeforest university hospital,Winston-Salem, USA | - | - | - | - | - | -- | - |
| Mount Sinai hospital, New York, USA | 3 | 3 | 0 | 0 | 1 | 0 | 10 (1.4) |
| Peritoneal malignancy institute, Basingstoke, UK | 0 | 32 | 0 | 0 | 0 | 0 | 32 (4.5) |
| St. George Hospital, Sydney, Australia | 10 | 13 | 12 | 2 | 1 | 2 | 40 (5.6) |
| INO, Rabat Morocco | 9 | 5 | 2 | 1 | 0 | 0 | 17 (2.4) |
| King Khaled Hospital, Najran, Saudi Arabia | 6 | 8 | 8 | 2 | 1 | 2 | 27 (3.8) |
|  | **`152 (21.4)** | **183 (25.8)** | **263 (37.1)** | **38 (5.3)** | **33 (4.6)** | **35 (4.9)** | **707*** |

*The primary tumour site for three patients treated at Mount Sinai Hospital was not known

Supplement S2

Primary sites of tumours classified as rare tumours

| Primary tumour site | No of patients |
| --- | --- |
| Ileum | 4 |
| Jejunum | 4 |
| Oesophagus | 1 |
| Uterus | 4 |
| Sarcoma | 6 |
| Hepatobiliary | 3 |
| Hepatoid carcinoma | 1 |
| Pancreas | 1 |
| Kidney | 1 |
| Leiomyomatosis | 1 |
| Not specified | 9 |

Supplement S3 Morphology of lesions in patients with colorectal PM

| **Morphological term** | **PCI region** | | | | | | | | | | | | | **Total N=2448 (%)** |
| --- | --- | --- | --- | --- | --- | --- | --- | --- | --- | --- | --- | --- | --- | --- |
|  | **0** | **1** | **2** | **3** | **4** | **5** | **6** | **7** | **8** | **9** | **10** | **11** | **12** |  |
| **Prespecified terms** | | | | | | | | | | | | | | |
| Adhesion | 0 | 0 | 0 | 0 | 0 | 0 | 0 | 1 | 0 | 0 | 0 | 0 | 1 | 2 (0.08) |
| Confluent disease | 8 | 11 | 2 | 7 | 4 | 13 | 28 | 11 | 5 | 0 | 0 | 1 | 5 | 95 (3.8) |
| Normal peritoneum | 48 | 70 | 88 | 80 | 72 | 46 | 36 | 65 | 53 | 78 | 82 | 81 | 71 | 870 (35.5) |
| Omental cake | 10 | 0 | 0 | 0 | 0 | 1 | 0 | 0 | 0 | 0 | 0 | 0 | 0 | 11 (0.4) |
| Plaque | 1 | 0 | 1 | 1 | 3 | 1 | 0 | 1 | 1 | 0 | 0 | 0 | 1 | 10 (0.4) |
| Thickening | 3 | 1 | 1 | 1 | 2 | 1 | 2 | 1 | 1 | 0 | 0 | 0 | 1 | 14 (0.5) |
| Scarring | 0 | 0 | 0 | 0 | 0 | 0 | 0 | 0 | 0 | 0 | 0 | 0 | 0 | 0 (0.0) |
| Tumor nodules | 48 | 27 | 21 | 17 | 37 | 48 | 46 | 28 | 43 | 31 | 29 | 30 | 33 | 438 (17.8) |
| **Unspecified terms** | | | | | | | | | | | | | | |
| Cyst | 0 | 0 | 0 | 0 | 0 | 0 | 0 | 0 | 0 | 0 | 0 | 0 | 0 | 0 (0.0) |
| Scalloping | 0 | 0 | 0 | 0 | 0 | 0 | 0 | 0 | 0 | 0 | 0 | 0 | 0 | 0 (0.0) |
| Mass | 0 | 0 | 0 | 0 | 0 | 0 | 0 | 0 | 0 | 0 | 0 | 0 | 0 | 0 (0.0) |
| Mucinous deposits | 0 | 0 | 0 | 1 | 1 | 1 | 2 | 1 | 1 | 1 | 1 | 0 | 0 | 9 (0.3) |
| Retraction | 0 | 0 | 0 | 0 | 0 | 0 | 0 | 0 | 0 | 0 | 0 | 0 | 0 | 0 (0.0) |
| Granulations | 0 | 0 | 0 | 0 | 0 | 0 | 0 | 0 | 0 | 0 | 0 | 0 | 0 | 0 (0.0) |

Supplement S4 Morphology of peritoneal lesions in patients with rare tumors

| **Morphological term** | **PCI region** | | | | | | | | | | | | | **Total N=287 (%)** |
| --- | --- | --- | --- | --- | --- | --- | --- | --- | --- | --- | --- | --- | --- | --- |
|  | **0** | **1** | **2** | **3** | **4** | **5** | **6** | **7** | **8** | **9** | **10** | **11** | **12** |  |
| **Prespecified terms** | | | | | | | | | | | | | | |
| Adhesion | 0 | 0 | 0 | 0 | 0 | 0 | 0 | 0 | 0 | 0 | 0 | 0 | 0 | 0 (0.0) |
| Confluent disease | 2 | 3 | 1 | 2 | 0 | 0 | 3 | 2 | 1 | 0 | 0 | 0 | 0 | 14 (4.8) |
| Normal peritoneum | 7 | 9 | 14 | 14 | 14 | 13 | 10 | 10 | 11 | 15 | 17 | 17 | 15 | 166 (57.8) |
| Omental cake | 1 | 0 | 0 | 0 | 0 | 0 | 0 | 0 | 0 | 0 | 0 | 0 | 0 | 1 (0.3) |
| Plaque | 0 | 0 | 0 | 0 | 0 | 1 | 0 | 1 | 0 | 0 | 0 | 0 | 0 | 2 (1.0) |
| Thickening | 0 | 0 | 0 | 0 | 0 | 0 | 0 | 0 | 0 | 0 | 0 | 0 | 0 | 0 (0.0) |
| Scarring | 0 | 0 | 0 | 0 | 0 | 0 | 0 | 0 | 0 | 0 | 0 | 0 | 0 | 0 (0.0) |
| Tumor nodules | 12 | 9 | 6 | 5 | 6 | 6 | 8 | 8 | 9 | 6 | 4 | 4 | 6 | 89 (31.0) |
| **Unspecified terms** | | | | | | | | | | | | | | |
| Cyst | 1 | 1 | 1 | 1 | 1 | 1 | 1 | 1 | 1 | 1 | 1 | 0 | 0 | 11 (3.8) |
| Scalloping | 0 | 0 | 0 | 0 | 0 | 0 | 0 | 0 | 0 | 0 | 0 | 0 | 0 | 0 (0.0) |
| Mass | 1 | 0 | 0 | 0 | 1 | 1 | 1 | 0 | 0 | 0 | 0 | 0 | 0 | 4 (1.3) |
| Mucinous deposits | 0 | 0 | 0 | 0 | 0 | 0 | 0 | 0 | 0 | 0 | 0 | 0 | 0 | 0 (0.0) |
| Retraction | 0 | 0 | 0 | 0 | 0 | 0 | 0 | 0 | 0 | 0 | 0 | 0 | 0 | 0 (0.0) |
| Granulations | 0 | 0 | 0 | 0 | 0 | 0 | 0 | 0 | 0 | 0 | 0 | 0 | 0 | 0 (0.0) |

Supplement S5 Morphology of peritoneal lesions in patients with peritoneal mesothelioma

| **Morphological term** | **PCI region** | | | | | | | | | | | | | **Total N=379 (%)** |
| --- | --- | --- | --- | --- | --- | --- | --- | --- | --- | --- | --- | --- | --- | --- |
|  | **0** | **1** | **2** | **3** | **4** | **5** | **6** | **7** | **8** | **9** | **10** | **11** | **12** |  |
| **Prespecified terms** | | | | | | | | | | | | | | |
| Adhesion | 0 | 0 | 0 | 0 | 0 | 0 | 0 | 0 | 0 | 0 | 0 | 0 | 0 | 0 (0.0) |
| Confluent disease | 2 | 3 | 3 | 3 | 3 | 4 | 5 | 6 | 3 | 1 | 1 | 1 | 1 | 36 (9.4) |
| Normal peritoneum | 8 | 8 | 11 | 15 | 12 | 10 | 7 | 12 | 11 | 15 | 12 | 14 | 14 | 149 (39.3) |
| Omental cake | 3 | 0 | 0 | 0 | 0 | 0 | 0 | 0 | 0 | 0 | 0 | 0 | 0 | 3 (0.7) |
| Plaque | 1 | 0 | 0 | 0 | 0 | 0 | 1 | 0 | 0 | 0 | 0 | 0 | 0 | 2 (0.5) |
| Thickening | 0 | 1 | 0 | 0 | 0 | 0 | 0 | 0 | 0 | 0 | 0 | 0 | 0 | 1 (0.2) |
| Scarring | 0 | 0 | 0 | 0 | 0 | 0 | 0 | 0 | 0 | 0 | 0 | 0 | 0 | 0 (0.0) |
| Tumor nodules | 12 | 15 | 14 | 10 | 13 | 10 | 11 | 9 | 15 | 12 | 15 | 13 | 12 | 161(42.4) |
| **Unspecified terms** | | | | | | | | | | | | | | |
| Cyst | 3 | 1 | 0 | 1 | 3 | 4 | 5 | 2 | 1 | 1 | 1 | 1 | 2 | 25 (6.5) |
| Scalloping | 0 | 0 | 0 | 0 | 0 | 0 | 0 | 0 | 0 | 0 | 0 | 0 | 0 | 0 (0.0) |
| Mass | 0 | 0 | 0 | 0 | 0 | 0 | 1 | 0 | 0 | 0 | 0 | 0 | 0 | 1 (0.2) |
| Mucinous deposits | 0 | 0 | 0 | 0 | 0 | 0 | 0 | 0 | 0 | 0 | 0 | 0 | 0 | 0 (0.0) |
| Retraction | 0 | 0 | 0 | 0 | 0 | 0 | 0 | 0 | 0 | 0 | 0 | 0 | 0 | 0 (0.0) |
| Granulations | 0 | 0 | 0 | 0 | 0 | 0 | 0 | 0 | 0 | 0 | 0 | 0 | 0 | 0 (0.0) |
| Pseudonodules | 1 | 0 | 0 | 0 | 0 | 0 | 0 | 0 | 0 | 0 | 0 | 0 | 0 | 1 (0.2) |

Supplement S6 Morphology of peritoneal lesions in patients with ovarian cancer

| **Morphological term** | **PCI region** | | | | | | | | | | | | | **Total N=2673 (%)** |
| --- | --- | --- | --- | --- | --- | --- | --- | --- | --- | --- | --- | --- | --- | --- |
|  | **0** | **1** | **2** | **3** | **4** | **5** | **6** | **7** | **8** | **9** | **10** | **11** | **12** |  |
| **Prespecified terms** | | | | | | | | | | | | | | |
| Adhesion | 1 | 2 | 0 | 1 | 0 | 0 | 1 | 2 | 2 | 0 | 0 | 1 | 1 | 10 (0.3) |
| Confluent disease | 18 | 60 | 21 | 21 | 15 | 41 | 69 | 30 | 15 | 5 | 3 | 3 | 9 | 310 (11.5) |
| Normal peritoneum | 68 | 53 | 87 | 85 | 99 | 56 | 37 | 53 | 81 | 130 | 126 | 125 | 98 | 1098 (41.0) |
| Omental cake | 35 | 0 | 0 | 0 | 0 | 0 | 0 | 0 | 0 | 0 | 0 | 0 | 0 | 35 (1.3) |
| Plaque | 2 | 7 | 0 | 1 | 2 | 2 | 3 | 3 | 1 | 0 | 0 | 0 | 0 | 21 (0.7) |
| Thickening | 2 | 1 | 0 | 0 | 1 | 0 | 4 | 0 | 0 | 0 | 0 | 0 | 0 | 8 (0.2) |
| Scarring | 0 | 4 | 0 | 1 | 2 | 1 | 3 | 1 | 1 | 0 | 0 | 0 | 1 | 14 (0.5) |
| Tumor nodules | 94 | 83 | 86 | 90 | 88 | 105 | 99 | 113 | 107 | 66 | 74 | 72 | 91 | 1168 (43.6) |
| **Unspecified terms** | | | | | | | | | | | | | | |
| Cyst | 0 | 1 | 1 | 0 | 0 | 0 | 0 | 0 | 0 | 0 | 0 | 0 | 0 | 2 (0.07) |
| Scalloping | 0 | 0 | 0 | 0 | 0 | 0 | 0 | 0 | 0 | 0 | 0 | 0 | 0 | 0 (0.0) |
| Mass | 0 | 0 | 0 | 0 | 0 | 0 | 0 | 0 | 0 | 0 | 0 | 0 | 0 | 0 (0.0) |
| Mucinous deposits | 0 | 0 | 0 | 0 | 0 | 0 | 0 | 0 | 0 | 0 | 0 | 0 | 0 | 0 (0.0) |
| Retraction | 1 | 0 | 0 | 0 | 0 | 0 | 0 | 0 | 0 | 0 | 0 | 0 | 0 | 1 (0.03) |
| Granulations | 0 | 0 | 0 | 0 | 0 | 0 | 0 | 0 | 0 | 0 | 0 | 0 | 0 | 0 (0.0) |
| Pseudonodules | 0 | 1 | 0 | 0 | 1 | 0 | 0 | 1 | 1 | 0 | 0 | 0 | 0 | 4 (0.1) |
| Induration | 0 | 0 | 0 | 0 | 0 | 0 | 0 | 1 | 0 | 0 | 0 | 1 | 0 | 2 (0.07) |

Supplement S7 Morphology of peritoneal lesions in patients with gastric PM

| **Morphological term** | **PCI region** | | | | | | | | | | | | | **Total N=359 (%)** |
| --- | --- | --- | --- | --- | --- | --- | --- | --- | --- | --- | --- | --- | --- | --- |
|  | **0** | **1** | **2** | **3** | **4** | **5** | **6** | **7** | **8** | **9** | **10** | **11** | **12** |  |
| **Prespecified terms** | | | | | | | | | | | | | | |
| Adhesion | 0 | 0 | 0 | 0 | 0 | 0 | 0 | 0 | 0 | 0 | 0 | 0 | 0 | 0 (0.0) |
| Confluent disease | 1 | 0 | 0 | 2 | 0 | 0 | 1 | 1 | 2 | 2 | 0 | 0 | 0 | 9 (2.5) |
| Normal peritoneum | 12 | 18 | 14 | 12 | 23 | 21 | 16 | 21 | 19 | 23 | 25 | 23 | 21 | 248 (69.0) |
| Omental cake | 0 | 0 | 0 | 0 | 0 | 0 | 0 | 0 | 0 | 0 | 0 | 0 | 0 | 0 (0.0) |
| Plaque | 2 | 0 | 1 | 0 | 0 | 0 | 0 | 0 | 0 | 0 | 0 | 0 | 0 | 3 (0.8) |
| Thickening | 0 | 0 | 0 | 0 | 0 | 0 | 0 | 0 | 0 | 0 | 0 | 0 | 0 | 0 (0.0) |
| Scarring | 1 | 1 | 0 | 1 | 1 | 1 | 1 | 1 | 1 | 0 | 0 | 0 | 1 | 9 (2.5) |
| Tumor nodules | 12 | 7 | 13 | 11 | 3 | 5 | 9 | 7 | 5 | 2 | 2 | 4 | 5 | 85 (23.6) |
| **Unspecified terms** | | | | | | | | | | | | | | |
| Cyst | 0 | 0 | 0 | 0 | 0 | 0 | 0 | 0 | 0 | 0 | 0 | 0 | 0 | 0 (0.0) |
| Scalloping | 0 | 0 | 0 | 0 | 0 | 0 | 0 | 0 | 0 | 0 | 0 | 0 | 0 | 0 (0.0) |
| Mass | 2 | 0 | 1 | 0 | 0 | 0 | 1 | 0 | 0 | 0 | 0 | 0 | 0 | 4 (1.1) |
| Mucinous deposits | 0 | 0 | 1 | 0 | 0 | 0 | 0 | 0 | 0 | 0 | 0 | 0 | 0 | 1 (0.2) |
| Retraction | 0 | 0 | 0 | 0 | 0 | 0 | 0 | 0 | 0 | 0 | 0 | 0 | 0 | 0 (0.0) |
| Granulations | 0 | 0 | 0 | 0 | 0 | 0 | 0 | 0 | 0 | 0 | 0 | 0 | 0 | 0 (0.0) |
| Pseudonodules | 0 | 0 | 0 | 0 | 0 | 0 | 0 | 0 | 0 | 0 | 0 | 0 | 0 | 0 (0.0) |
| Induration | 0 | 0 | 0 | 0 | 0 | 0 | 0 | 0 | 0 | 0 | 0 | 0 | 0 | 0 (0.0) |

Supplment S8 Morphology of peritoneal lesions in patients with appendiceal tumors and PMP

| **Morphological term** | **PCI region** | | | | | | | | | | | | | **Total N=1663 (%)** |
| --- | --- | --- | --- | --- | --- | --- | --- | --- | --- | --- | --- | --- | --- | --- |
|  | **0** | **1** | **2** | **3** | **4** | **5** | **6** | **7** | **8** | **9** | **10** | **11** | **12** |  |
| **Prespecified terms** | | | | | | | | | | | | | | |
| Adhesion | 0 | 0 | 1 | 0 | 0 | 0 | 0 | 0 | 0 | 0 | 0 | 0 | 1 | 2 (0.1) |
| Confluent disease | 36 | 37 | 26 | 29 | 19 | 19 | 37 | 38 | 27 | 12 | 12 | 13 | 17 | 322 (19.3) |
| Normal peritoneum | 23 | 20 | 48 | 48 | 52 | 33 | 15 | 31 | 38 | 64 | 70 | 68 | 50 | 560 (33.6) |
| Omental cake | 7 | 0 | 0 | 0 | 0 | 0 | 0 | 0 | 0 | 0 | 0 | 0 | 0 | 7 (0.4) |
| Plaque | 6 | 8 | 4 | 6 | 6 | 5 | 2 | 1 | 3 | 2 | 0 | 0 | 2 | 45 (2.7) |
| Thickening | 0 | 0 | 0 | 0 | 1 | 0 | 1 | 2 | 2 | 0 | 0 | 0 | 0 | 6 (0.3) |
| Scarring | 1 | 0 | 1 | 0 | 0 | 0 | 0 | 1 | 2 | 1 | 0 | 0 | 1 | 6 (0.3) |
| Tumor nodules | 58 | 60 | 42 | 42 | 46 | 66 | 68 | 52 | 55 | 43 | 42 | 41 | 51 | 666 (40.3) |
| **Unspecified terms** | | | | | | | | | | | | | | |
| Cyst | 0 | 0 | 0 | 0 | 0 | 0 | 0 | 0 | 0 | 0 | 0 | 0 | 0 | 0 (0.0) |
| Scalloping | 0 | 0 | 0 | 0 | 0 | 0 | 0 | 1 | 1 | 1 | 0 | 1 | 1 | 5 (0.3) |
| Mass | 0 | 0 | 0 | 0 | 0 | 0 | 0 | 0 | 0 | 0 | 0 | 0 | 0 | 0 (0.0) |
| Mucinous deposits | 4 | 4 | 5 | 3 | 4 | 4 | 1 | 2 | 3 | 3 | 2 | 3 | 6 | 44 (2.6) |
| Retraction | 0 | 0 | 0 | 0 | 0 | 0 | 0 | 0 | 0 | 0 | 0 | 0 | 0 | 0 (0.0) |
| Granulations | 0 | 0 | 0 | 0 | 0 | 0 | 0 | 0 | 0 | 0 | 0 | 0 | 0 | 0 (0.0) |
| Pseudonodules | 0 | 0 | 0 | 0 | 0 | 0 | 0 | 0 | 0 | 0 | 0 | 0 | 0 | 0 (0.0) |
| Induration | 0 | 0 | 0 | 0 | 0 | 0 | 0 | 0 | 0 | 0 | 0 | 0 | 0 | 0 (0.0) |

Supplement S9 Figure 13- region distribution of the morphological term ‘normal peritoneum’


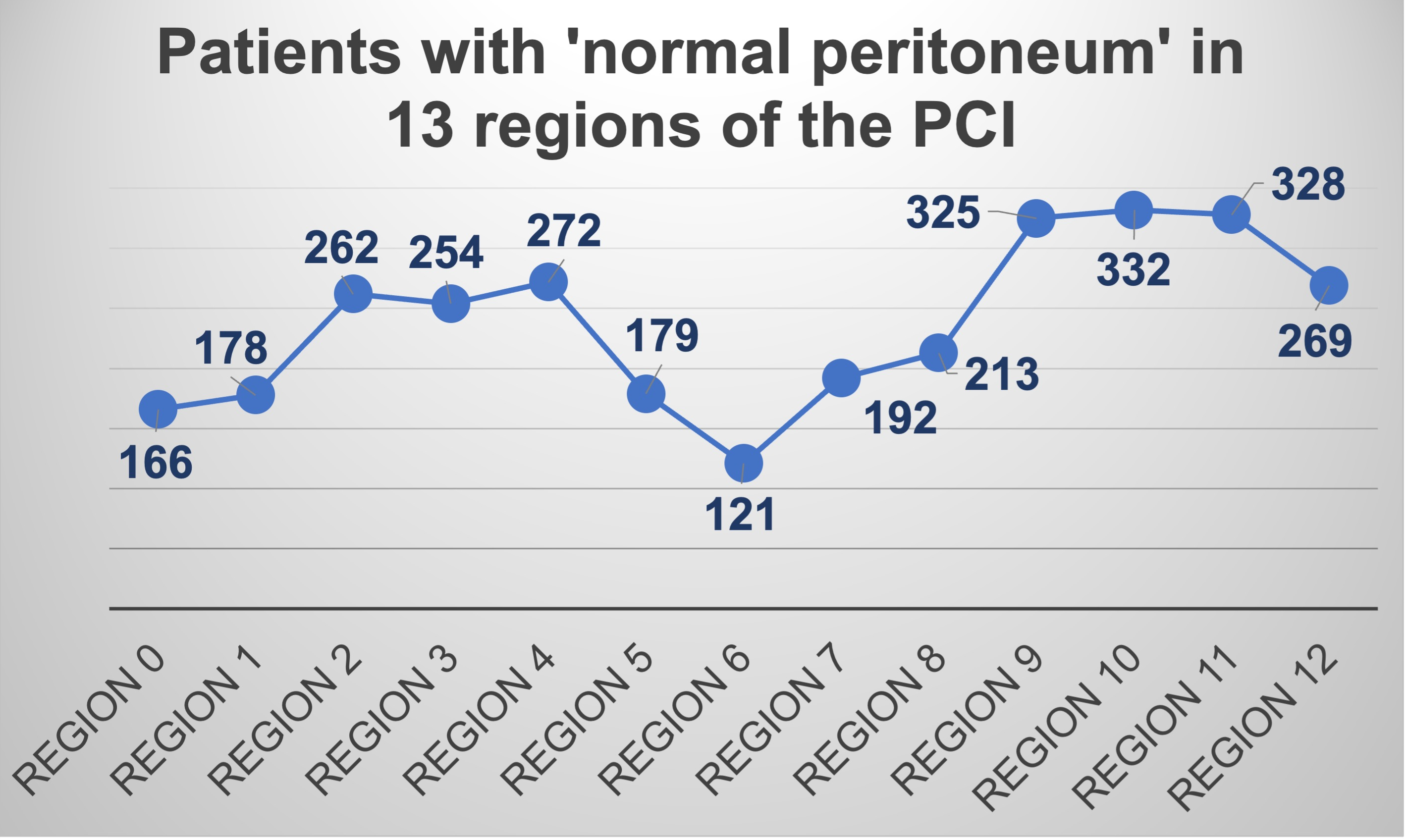


Supplement S10 Figure 13 region distribution of microscopic (occult) disease in ‘normal appearing’ peritoneum


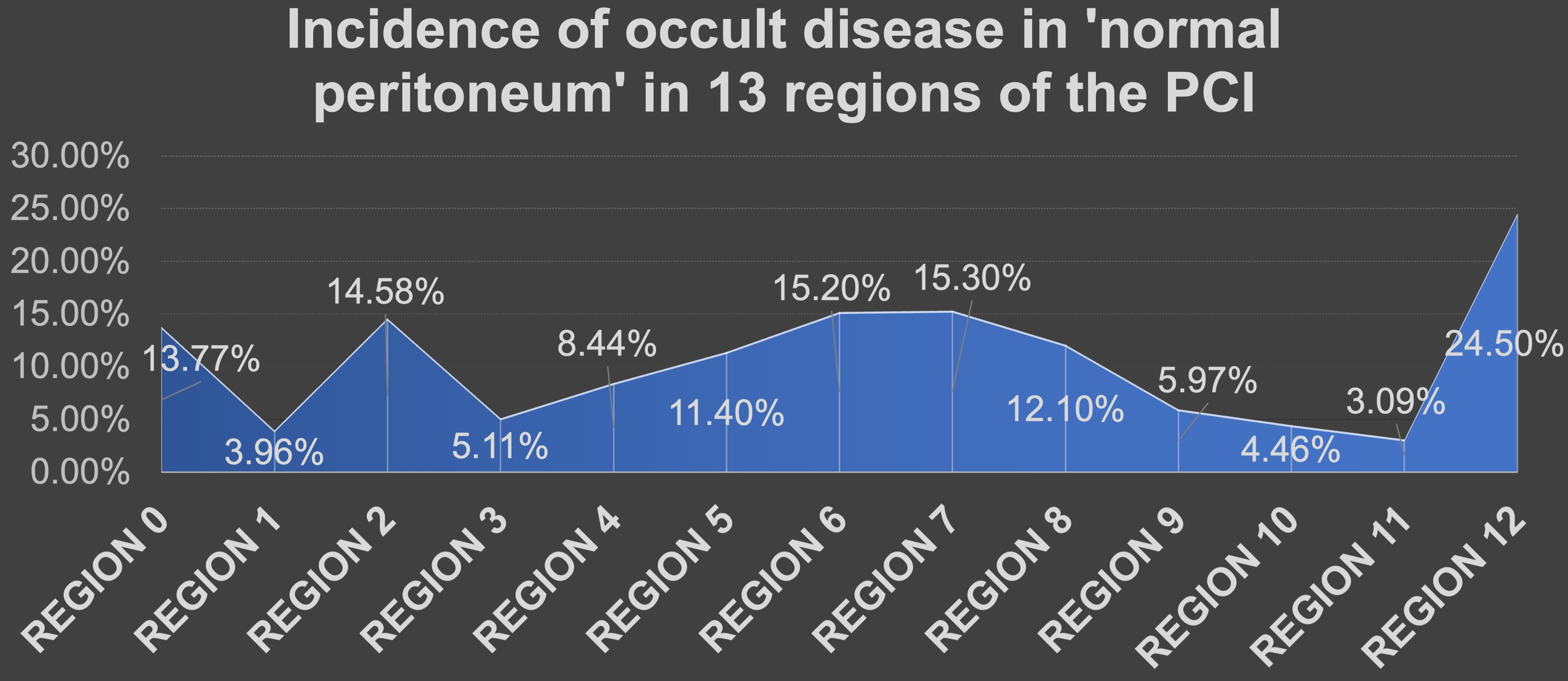

Supplement: Supplementary file 1 — Supplementary file1 (DOCX 23980 KB) [file 10434_2024_16035_MOESM1_ESM.docx]
